# CT scan evaluation of glenoid component fixation: a prospective study of 27 minimally cemented shoulder arthroplasties

**DOI:** 10.1007/s00590-012-1126-5

**Published:** 2012-11-09

**Authors:** A. Vidil, Ph. Valenti, F. Guichoux, J. H. Barthas

**Affiliations:** 1 Hôpital Saint Joseph, 185, rue Raymond Losserand, 75014 Paris, France; 2 Clinique Jouvenet 6, Square Jouvenet, 75016 Paris, France

**Keywords:** Shoulder arthroplasty, Pegged glenoid component, Bone ingrowth, CT scan, Radiolucency

## Abstract

**Background:**

Glenoid component failure is the most common complication of total shoulder arthroplasty. It can be correlated with failure of the component itself to resist wear and deformation, failure of fixation or failure of the glenoid bone. Anchor Peg Glenoid component (Depuy^®^) seems to have a higher bone fixation in biomechanical canine model: it is a all-polyethylene, concave component with one circumferentially fluted, central, interference-fit peg and three small cemented peripheral pegs.

**Materials and methods:**

We realized a prospective study of Anchor Peg total shoulder arthroplasty, included 27 patients suffering from primary arthrosis or arthritis, without rotator cuff tear. A clinical and radiographic evaluation was performed at 3 months, 1 and 2 years; a CT scan was made in postoperative and analyzed central peg’s bone integration 1 year later.

**Results:**

Improvement of postoperative Constant score and radiographic good results were correlated with satisfactory subjective results reported by patients. We observed radiolucent lines under glenoid component in 3 cases. Twenty-six CT scans were available at 1 year: it showed complete bone integration around the central peg in 21 cases and partial peripheral bone integration in four cases. Only one patient had any tissue integration around the peg, probably because of his implantation near cortical bone of scapular spine.

**Discussion/conclusion:**

Long-term result of arthroplasty is correlated with glenoid durable fixation to underlying bone: this study shows higher fixation of glenoid component with bone integration of central peg. However, these results will have to be confirmed in a later revision.

## Introduction

Glenoid loosening continues to be the primary cause of failure of total shoulder arthroplasty. In traditional cemented glenoid components, radiolucent lines at the bone cement interface of glenoid stems are common. The appearance or progression of these radiolucencies may coincide with symptomatic component loosening [1].

From this observation, new concepts and innovations have been proposed to enhance glenoid fixation and durability.

Metal-back glenoid component studies revealed osteolysis, clinical loosening and component failure with long-term follow-up [2]. Consequently, cemented all-polyethylene components were focused upon; both experimental and clinical evidences have shown that polyethylene components with biaxial pegs demonstrate superior fixation and a decreased rate of early glenoid component loosening compared with keeled implants [3–5].

**Fig. 1:**
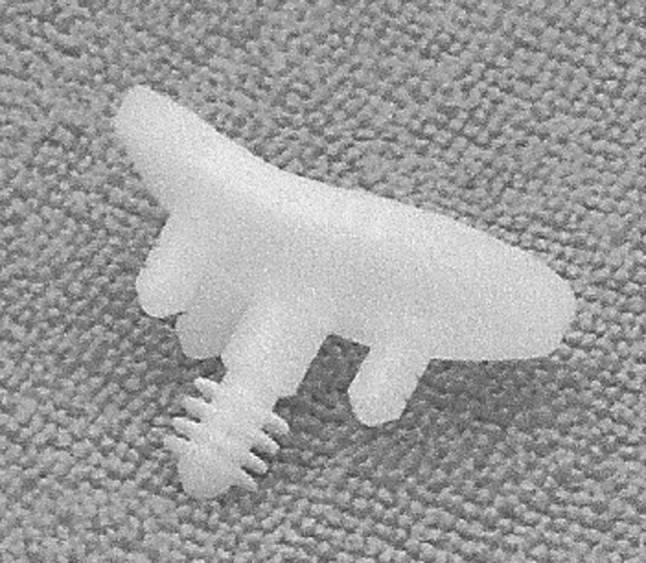
Depuy^®^ Anchor Peg glenoid component

For 10 years, a novel pegged, all-polyethylene glenoid component was developed. It features a circumferentially fluted, central, interference-fit peg for tissue integration and 3 small cemented peripheral pegs (Fig. 1). The concept has been validated with in vivo canine study. It demonstrated that use of a glenoid component with a flanged central peg results in superior mean fixation strength in a weight-bearing canine model at short-term follow-up [6]. Radiographic and histologic examination showed that the central fluted peg achieved bone ingrowth around in all cases.

The purpose of this study was to evaluate the bone integration of the flanged central peg, using CT scan to measure bone ingrowth, and to show a correlation with good clinical results and longevity of the glenoid component.

## Materials and methods

This series is a prospective consecutive study of 26 patients (27 shoulders), suffering from primary or secondary osteoarthritis, without rotator cuff tear, operated by the same surgeon between November 2005 and November 2009.

There were 17 women and 9 men included in this study; 1 woman had undergone bilateral shoulder arthroplasty. The mean age at the time of surgery was 66 (range 56–84 years old). Indications for arthroplasty were primary osteoarthritis in 18 shoulders, post-traumatic arthritis in 5 and rheumatoid arthritis in 3. One osteoarthritis shoulder had undergone a previous procedure with humeral component implantation; it remained painful because of a glenoid chondrolysis resulting in total shoulder prosthesis.

A clinical and radiographic evaluation was performed at 3 months, then at 1 and 2 years. Clinical assessments were focused on active elevation and external rotation; the global result was evaluated using absolute Constant score. The radiographic evaluation included an axillary lateral radiograph and an anteroposterior radiograph made perpendicular to the plane of the scapula.

A CT scan was made in postoperative and analyzed central peg’s bone integration 1 year later. It was carried out with very thin sections, <1 mm, and high-resolution reconstruction in oblique coronal and sagittal planes aligned to the glenoid (helical scans with 0.625-mm slice thickness and interval, 140 kV, automatic mAS). Due to humeral head’ artefacts, it was not possible to measure directly bone density and macroscopic bone integration was only taken into consideration.

**Fig. 2:**
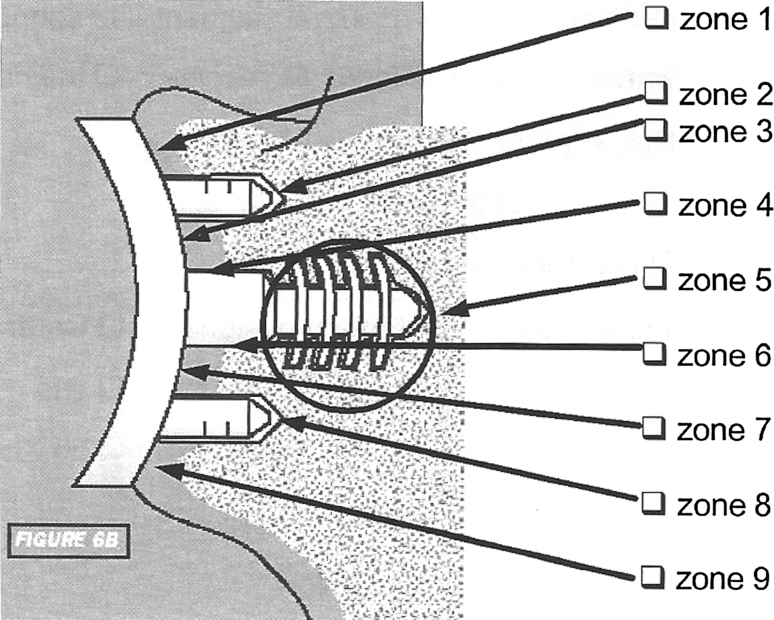
Glenoid radiolucencies’ areas

Both X-rays and CT scan were used to analyze glenoid version, humeral head position and radiolucent lines, which were located in 9 areas (Fig. 2).

One patient was lost to follow-up. The remaining 25 (26 shoulders) had a minimum of 2 years of follow-up and formed the study group. The mean duration of clinical follow-up was 4 years (range 2–7 years).

### Surgical technique

All arthroplasties were performed with the Anchor Peg glenoid component (Depuy, a Johnson & Johnson company, Warsaw, Indiana, USA) that featured a circumferentially fluted, interference-fit central peg for osseous integration and 3 small peripheral pegs designed for minimally cemented fixation. All 3 peripheral pegs were located 10.3 mm from the center of the implant at the 12 o’clock, 5 o’clock and 7 o’clock positions to maximize component stability.

A deltopectoral approach was used for exposure. Components were implanted following the manufacturer’s guidelines, which included the following steps regarding glenoid insertion.

The glenoid was prepared by removing any remaining labrum tissue for exposure. The center of the glenoid was chosen and prepared by creating a centering hole with an initial drill. The glenoid reamer was used to create a concentric glenoid articular surface for seating of the glenoid component and also to correct any excessive glenoid retroversion. Drill guides were used to precisely bore the central fixation hole and the smaller peripheral fixation holes. Each hole was palpated to its full depth with a probe and checked by direct visualization to determine whether it penetrated the glenoid vault cortex. The quality of the glenoid bone preparation was checked by inserting a trial glenoid component and verifying that it did not rock even when an eccentric load was applied to its rim.

All holes were irrigated to remove blood and dried. Bone cement was applied with a syringe, and bone graft from the drill holes was applied to the flutes of the central peg. The final prosthesis was then inserted and a glenoid impactor used to ensure seating of the prosthesis.

The humeral component was cemented in a mean of 20° of retroversion. The standard 6 mm of diametral mismatch was utilized in all arthroplasties.

## Results

### Clinical findings

The mean absolute Constant score improved from 27.5 (range 14–44) preoperatively to 74.5 (range 46–91) at the time of the latest follow-up. All shoulder motion measures improved significantly. Anterior elevation increased from 93° (range 50–140) preoperatively to 139° (range 90–170) at revision, and the external rotation improved from 12.5° (range 10–40) preoperatively to 49° (range 20–60) at the time of the latest follow-up. Preoperatively, the highest level that could be reached in internal rotation was the hip in 3 shoulders, the buttocks in 8, L5 in 10, and L1 in 6. At revision, the mean increase was 5 sacral and vertebral levels. Improvement of clinical criteria was correlated with satisfactory subjective results reported by patients.

### Radiographic analysis

Preoperatively, the arthritis was classified as Samilson 2 in 18 shoulders and 3 in 9. The glenoid morphology was graded as Walch A1 in 11 shoulders, A2 in 6, B1 in 7, and B2 in 3.

Early radiolucent lines were observed under glenoid component in three cases. It was located in area 7 and 9, <1 mm, and nonevolutive with follow-up in 2 shoulders. One patient had a more extensive radiolucent lines, in area 1, 4, 7 and 9, due to a bad cementation of the glenoid component. At revision, thickness was similar, without failure of the prosthesis.

### CT scan evaluation

Twenty-six CT scans were available at 1 year. Every glenoid component was in a neutral version, and the humeral head was centered with the glenoid in 24 cases. An anterior subluxation, <25 %, was observed in 2 shoulders, due to subscapularis insufficiency, correlated with a preoperative muscle degenerative infiltration without tendon’s tear.

Radiolucent lines’ analysis was more precise with CT scan, but a good correlation remained with radiographic evaluation.

**Fig. 3:**
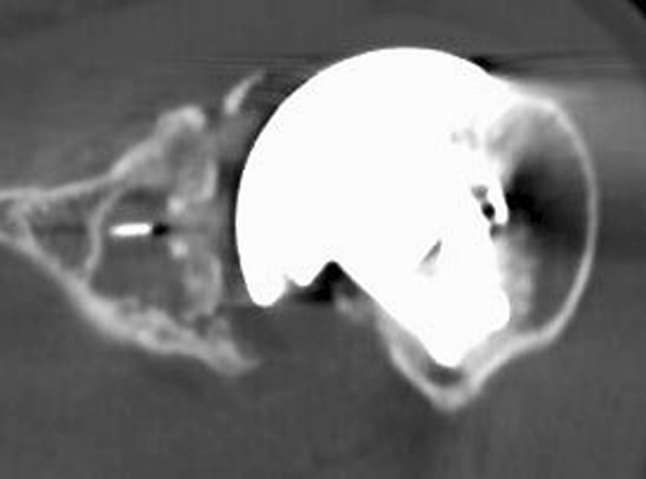
CT scan evaluation: no tissue integration around the central peg

Only one patient had no tissue integration around the central peg, due to a subchondral bone defect. However, a partial peripheral condensation was observed at the upper part of the peg (Fig. 3).

**Fig. 4:**
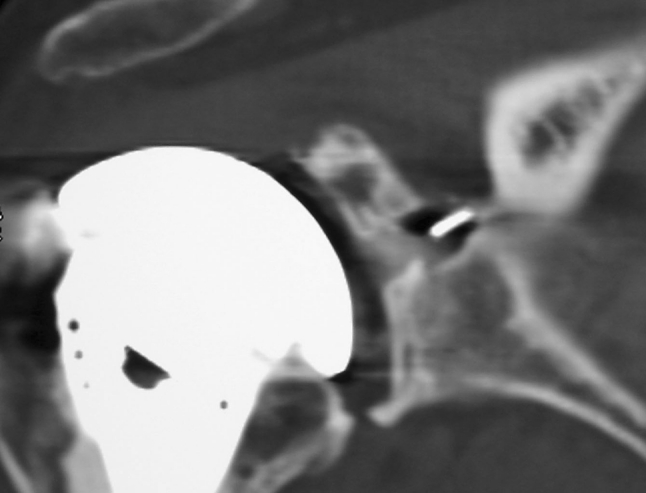
CT scan evaluation: peripheral bone integration of the central peg

Four patients had only a peripheral bone integration, without bone ingrowth around the flanges, probably because of his implantation near cortical bone of scapular spine (Fig. 4).

**Fig. 5:**
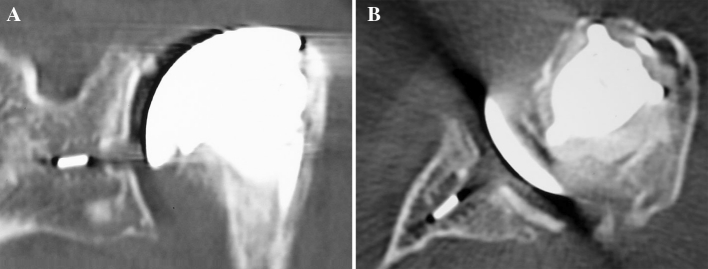
CT scan evaluation: bone ingrowth between the flanges of the central peg

In twenty-one shoulders, CT scan showed complete integration, with bone ingrowth around the peg flanges, as shown in canine model (Fig. 5).

### Complications and revisions

One patient had a postoperative capsulitis and remained with a low mobility at revision. The active elevation was 90° and the external rotation was 20°, but without any pain. One patient had a recurrence of pain after 3 years, correlated with an acromial metastasis of a prostate cancer. Any modification of the prosthesis was observed on X-rays. No glenoid component was radiographically loose and no shoulder has been revised.

## Discussion

Failure of the glenoid component is the most common complication of total shoulder arthroplasty and accounts for a majority of the unsatisfactory results after this procedure. The etiology of component loosening is multifactorial and is associated with the implant design and materials, the method of fixation, the surgical technique and patient factors such as bone loss and bone quality [7].

Emphasis is currently focused on contemporary cemented all-polyethylene components, and pegged design seems to provide the most durable fixation. In a multicenter study of 328 total shoulder arthroplasties, Lazarus et al. [8] reported superior technical outcomes for biaxially pegged components compared with keeled components. These authors provided several plausible explanations for this finding: the greater precision of the match between the geometry of the pegged component and that of the prepared glenoid bone, the precision of the instrumentation used with pegged component, and the smaller volume of cement used with pegged component, resulting in the generation of less heat and a lower risk of necrosis of adjacent bone.

Since 2010, four reports have described clinical and radiographic results of a glenoid component with both minimally cemented peripheral pegs and a central peg with flanges designed to permit bone ingrowth.

Churchill et al. [9] reviewed 20 total shoulder arthroplasties at a minimum follow-up of 5 years and reported bone ingrowth between the flanges of the central peg in 15 of the glenoid components. In Groh’s study, 83 patients were treated for primary shoulder osteoarthritis with joint replacement, using uncemented fluted pegged glenoid component [10]. At a minimum of 2 years’ follow-up, all glenoid components were assessed as having grade 0 radiolucency, and evidence of finger-like projections of bone between the flanges of the implant was found in 24 cases.

Arnold et al. [11] evaluated 35 total shoulder prosthesis at a mean follow-up of 43 months with use of computed tomography. The presence of bone between the flanges of the central peg of the glenoid was demonstrated in 32 of the 35 shoulders. More recently, Wirth et al. [12] analyzed clinical and radiographic outcomes of 44 shoulder replacements with a minimum of 2 years of follow-up. Twenty shoulders had perfect seating and radiolucency grades, 30 had increased radiodensity between the flanges of the central peg, and three demonstrated osteolysis. At the latest follow-up (range 4–7 years), all these series reported no shoulder revision for failure of glenoid component.

In a series of 11 patients, Trial et al. [3] studied the micromotion of this minimally cemented fluted pegged glenoid component by using radiostereometric analysis, which measures the position of rigid bodies in three dimensions. Two groups were identified: the first group showed little if any migration during the entire study period, and the second group showed large early rotation movement. CT scans confirmed that the first no migrating group with no focal lucencies had osseointegration around the central peg of the glenoid implant; in the migrating group, the focal lucencies observed in the plain radiographs were indicative of voids around the central peg where no bone was present.

They correlated the absence of osseointegration with the lack of immediate implant stability and with too early daily activities, involving the shoulder for lifting or holding a weight with an outstretched arm.

For many years, the primary method of evaluation of glenoid radiolucencies and loosening was plain radiography. In 2002, Lazarus et al. [8] described a radiographic classification system to evaluate pegged glenoid components for radiolucencies. Yian et al. [13] reported a CT scan study to evaluate pegged glenoid components utilizing 3 mm CT cuts and concluded that computed tomography scans were a more sensitive and reproducible tool for the assessment of loosening of pegged glenoid components than was fluoroscopically guided conventional radiography. However, these authors did note that some pegs were difficult to analyze due to artifact from the humeral component. Currently, the latest CT scans are carried out with 0.625 mm axial cuts as well as coronal and sagittal high-resolution reconstructions and allows an adequately evaluation of each peg.

Arnold et al. [11] analyzed 35 total shoulder arthroplasties by CT scan and found 23 shoulders with complete integration of the central peg, with bone ingrowth all around the peg flanges, and 3 shoulders without any tissue integration. From our own findings, we conclude the same as Arnold. CT scan should not be necessarily advocated for routine follow-up, even if it is an excellent (and more accurate) adjunct to plain radiographs for a more in-depth study of glenoid components.

## Conclusion

The early and intermediate-term results of this glenoid component are comparable with those reported in the literature for glenoid implants with multiple pegs that were all cemented. CT scan is a more sensitive and reproducible tool for evaluation of radiolucent lines at the bone cement interface; very thin sections and high-resolution reconstructions allow measurement and analysis of macroscopic bone integration around the central peg. Long-term follow-up will determine whether this bone ingrowth will be associated with the durability of the glenoid implant in total shoulder arthroplasty.
